# White matter structural and network topological changes in moyamoya disease with limb paresthesia: A study based on diffusion kurtosis imaging

**DOI:** 10.3389/fnins.2022.1029388

**Published:** 2022-10-31

**Authors:** Rujing Sun, Shi-Yu Zhang, Xu Cheng, Sangma Xie, Peng-Gang Qiao, Gong-Jie Li

**Affiliations:** ^1^Department of Radiology, Beijing Friendship Hospital, Capital Medical University, Beijing, China; ^2^School of Automation, Hangzhou Dianzi University, Hangzhou, China; ^3^Department of Radiology, Affiliated Hospital of Academy of Military Medical Sciences, Beijing, China

**Keywords:** moyamoya disease (MMD), magnetic resonance imaging (MRI), diffusion kurtosis imaging (DKI), brain network, limb paresthesia

## Abstract

**Purpose:**

To investigate the structural and network topological changes in the white matter (WM) in MMD patients with limb paresthesia by performing diffusion kurtosis imaging (DKI).

**Materials and methods:**

A total of 151 MMD patients, including 46 with left-limb paresthesia (MLP), 52 with right-limb paresthesia (MRP), and 53 without paresthesia (MWP), and 28 healthy controls (HCs) underwent whole-brain DKI, while the surgical patients were reexamined 3-4 months after revascularization. The data were preprocessed to calculate the fractional anisotropy (FA) and mean kurtosis (MK) values. Voxel-wise statistics for FA and MK images were obtained by using tract-based spatial statistics (TBSS). Next, the whole-brain network was constructed, and global and local network parameters were analyzed using graph theory. All parameters were compared among the HC, MWP, MLP, and MRP groups, and changes in the MMD patients before and after revascularization were also compared.

**Results:**

The TBSS analysis revealed significant reductions in FA and MK in extensive WM regions in the three patient groups. In comparison with the MWP group, the MLP group showed reductions in FA and MK in both right and left WM, mainly in the right WM, while the MRP group mainly showed a reduction in FA in the left WM region and demonstrated no significant change in MK. The graph theoretical analysis showed decreased global network efficiency, increased characteristic path length, and increased sigma in the MWP, MRP, and MLP groups in comparison with the HC group. Among local network parameters, the nodal efficiency decreased in the bilateral MFG and IFGtriang, while the degree decreased in the MFG.L and bilateral IFGtriang. Patients with right-limb paresthesia showed the lowest nodal efficiency and degree in MFG.L and IFGtriang.L, while those with left-limb paresthesia showed the lowest nodal efficiency in MFG.R and IFGtriang.R and the lowest degree in IFGtriang.R.

**Conclusion:**

A DKI-based whole-brain structural and network analysis can be used to detect changes in WM damage and network topological changes in MMD patients with limb paresthesia. FA is more sensitive than MK in detecting WM injury, while MFG and IFGtriang are the key nodes related to the development of acroparesthesia.

## Introduction

Moyamoya disease (MMD) is a cerebrovascular disease caused by the development of moyamoya vessels to compensate for chronic bilateral occlusion of the internal carotid arteries (Kuroda and Houkin, 2008; Kazumata et al., 2016a). Voxel-based morphometric analyses have recently shown extensive microstructural changes in the white matter (WM) of patients with MMD, probably due to nerve function impairment (Kazumata et al., 2015). We had previously used diffusion kurtosis imaging (DKI) and region of interest (ROI) analyses to identify changes in the DKI parameters in the WM regions associated with sensory transduction pathways in MMD patients with acroparesthesia (Qiao et al., 2020). However, ROI- or voxel-based morphometry can only identify the WM region involved and cannot identify the affected neural pathways.

Complex models of the brain network consisting of neural units (e.g., neurons and brain regions) with structural or functional connections have been constructed in many recent studies (Bullmore and Sporns, 2009; Rubinov and Sporns, 2010; Van Essen et al., 2012; Liao et al., 2017). In these studies, non-invasive neuroimaging techniques (Bullmore and Sporns, 2009; Yong and Evans, 2010; van den Heuvel and Sporns, 2013) such as multimodal magnetic resonance imaging (e.g., structural MRI, diffusion MRI, and functional MRI) were combined with graph theoretical approaches to map the structural and functional connectivity patterns in the human brain, improving our understanding of the topological properties of the complex brain network.

Small-world architecture has shown increasing relevance in descriptions of the human brain network because of the associated low wiring and energy costs and effective information segregation and integration (Watts and Strogatz, 1998). In this regard, changes in the small-world characteristics of the brain network have been shown to be associated with normal aging and brain diseases such as neurodegenerative diseases, epilepsy, gliomas, and schizophrenia (Lo et al., 2010; van den Heuvel et al., 2010; Griffa et al., 2013; Huang et al., 2014). However, only a few studies have evaluated the brain network in patients with MMD, and most of them were diffusion tensor imaging (DTI)-based correlation analyses. DTI is constrained by technical insufficiencies: It is based on the assumption that water molecules diffuse freely and that diffusion can be characterized by a Gaussian distribution. Moreover, the tensor model is based on the observation that water diffusion is anisotropic in many tissues. Thus, this model performs well in regions where fibers are aligned along a single axis and fails in regions with several fiber populations aligned along intersecting axes because it cannot simultaneously map several diffusion maxima. DKI is an extension of DTI that estimates both the diffusion and kurtosis tensors in order to characterize non-Gaussian diffusion dynamics within complex biological tissue (van den Heuvel et al., 2010; Kazumata et al., 2016a). The Orientation Distribution Function (ODF) assessments based on DKI is one of the important methods to describe the directionality of multimodal diffusion in regions with complex fiber architecture present in brain and other biological tissues, which can resolve fiber crossings in a model independent manner (Lazar et al., 2008; Glenn et al., 2015). Therefore, in this study, we constructed a DKI-based brain structural network of MMD patients with acroparesthesia and used graph theory to characterize the topological organization of the brain structural network, with the aim of understanding the central mechanism of acroparesthesia in MMD patients and thereby facilitating accurate assessment and clinical treatment of MMD.

## Materials and methods

### Participants

Between January 2015 to July 2018, a total of 151 patients with MMD, including 46 with left-limb paresthesia (MLP group, age = 36.7 ± 9.7 years; 24 males), 52 with right-limb paresthesia (MRP group, age = 37.5 ± 9.8 years; 30 males), and 53 without paresthesia (MWP group, age = 39.1 ± 7.0 years; 26 males), were recruited in this study. Of these, 18 patients in the MLP group, 17 in the MRP group, and 24 in the MWP group underwent encephaloduroarteriosynangiosis (EDAS). Limb paresthesia manifested as paroxysmal limb numbness or hypoesthesia. In addition, 28 age- and sex-matched healthy volunteers were recruited as healthy controls (HC group).

The inclusion criteria were as follows: (1) diagnosis and staging using digital subtraction angiography (DSA); (2) stable condition, no cerebral hemorrhage before scanning, no cerebral infarction on conventional MRI, and cooperation for the examination; (3) no other neurological or psychiatric disorder; and (4) right-handedness. All patients underwent MRI within 1 week before and after DSA, and the 59 surgical patients underwent a second MRI 3–4 months after EDAS. All volunteers were informed of the procedures and precautions before examination and signed an informed consent form before participating in the study. The ethics committee of ^***^ approved the study.

### Physiological and biochemical tests

Before MRI data acquisition, a series of physiological and biochemical tests were conducted in all of the patients to measure their blood pressure, blood sugar level, and total cholesterol level. In addition, data pertaining to their smoking and drinking histories were collected.

### Image acquisition

A Siemens 3.0-Tesla Skyra magnetic resonance imaging scanner (Siemens AG, Erlangen, Germany) with a 32-channel standard head coil was used to acquire images. All volunteers and patients with MMD underwent whole-brain DKI, which was performed using an axial echo-planar imaging sequence with the following parameters: repetition time (TR), 5,600 ms; echo time (TE), 92 ms; field of view (FOV), 228 × 228 mm^2^; matrix, 76 × 76; slice thickness, 3 mm; number of slices, 33; and b value = 0, 1,000, and 2,000 s/mm2; the diffusion sensitive gradient field was applied in 30 directions.

### Diffusion magnetic resonance imaging data preprocessing

Data quality was checked initially, and participants with serious data quality problems on visual inspection were excluded. Then, data were converted from DICOM format to nifti format using dcm2nii. Diffusion MRI data were preprocessed using the FMRIB software library (FSL, version 6.0.3)^1^. Data preprocessing involved the following steps: head-motion/eddy-current correction (“eddy_correct” command); gradient direction correction (“fdt_rotate_bvecs” command); and brain mask extraction (“bet2” command). Finally, the fractional anisotropy (FA), axial diffusivity (AD), radial diffusivity (RD), and mean diffusivity (MD) values were calculated using the “dtifit” command of FDT in FSL, while the mean kurtosis (MK), axial kurtosis (AK), and radial kurtosis (RK) values were calculated using DKE software. Only images with b = 0 and 1,000 s/mm2 were employed for DTI fitting, and all the data (b = 0, 1,000, 2,000 s/mm2) were used for DKI fitting.

### Tract-based spatial statistics

A voxel-wise statistical analysis of FA and MK images was performed using the TBSS toolbox in FSL. We used flirt and fnirt to linearly and then non-linearly register the individual FA image to the FMRIB58 FA template image in the standard space. The threshold average FA for the WM skeleton was set to 0.2, and the region with an FA value of > 0.20 was considered as the final skeleton region. Finally, an independent FA skeleton image was generated in the standard space. After execution of all TBSS steps on the basis of FA values, the TBSS analysis was performed on MK values using the “tbss_non_FA” command to obtain MK values for all skeletons tested.

### Whole-brain network construction

The preprocessed data were used for the reconstruction or calculation of the orientation distribution function in the DKE software^2^. The fiber-tracking module in the DKE software was used for whole-brain fiber tracking with the following parameters: FA threshold, 0.1; angular threshold, 45°; length threshold, 20 mm; step size, 1 mm; and number of random seed points, 10^5^ (default parameter of DKE).

Linear and non-linear registration tools (flirt and fnirt, respectively) were used to register the 90 brain regions of the automated anatomic labeling (AAL) template into the individual diffusion space to define network nodes. First, we employed the linear method to register the native FA map to the individual brain T1 image in T1 space by using the registration tool flirt (Jenkinson et al., 2002). Then, we used flirt and fnirt to linearly and then non-linearly register the individual brain T1 image to the MNI152_T1_2-mm_brain image in the standard space. Next, we inverted the two derived transformations from the diffusion MRI space to the T1 space and from the T1 space to standard space. Finally, the two inverted transformations were applied to warp the AAL from the standard space to the native diffusion MRI space.

**FIGURE 1:**
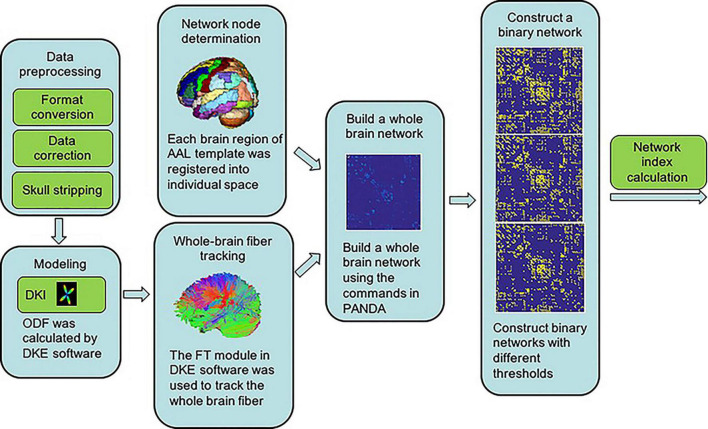
Schematic diagram outlining the construction of the brain network.

A whole-brain network was then constructed using the command g_Deterministic Network in PANDA, with AAL brain regions as nodes and the number of fibers connecting brain regions as the weights of the edges (Figure 1).

### Graph theoretical analysis

Binary networks with thresholds of 1, 2, and 3 were constructed for calculation of network indices (global and local network parameters). We calculate the area under the curve (AUC) for each network measure (threshold = 1, 2, 3) to provide a scalar, which does not depend on the specific threshold selection (Wang et al., 2015). Global network parameters included global efficiency, local efficiency, clustering coefficient (CC), normalized CC (γ = CC/CCrand), sigma, and characteristic path length. Local network parameters were also used to describe the local network characteristics of each brain region in the structural network, and these parameters included degree, shortest path length, local efficiency, nodal efficiency, CC, and betweenness centrality (Rubinov and Sporns, 2010). All network analyses were performed using the Graph Theoretical Network Analysis (GRETNA) toolbox (Wang et al., 2015).

### Statistical analysis

An analysis of covariance was performed on FA and MK images of the skeleton using the “randomize” command in FSL (50,000 permutations) to identify differential regions among groups. Within differential regions, a *post hoc* test was performed for pairwise comparisons of two groups using the “randomize” command in FSL (50,000 permutations) to identify differential regions (patterns) between groups. The family-wise error (FWE) method was used for multiple comparison correction in all statistical analyses (*p* < 0.05). The JHU ICBM white matter atlas was then used to localize and label regions showing significant differences between groups. ANOVA followed by *post hoc* tests was performed to identify differences in network indices among the four groups. Statistical analyses were corrected for multiple comparisons using the FWE method with thresholds of *p* < 0.05 and *p* < 0.01.

## Results

### Demographic data of the study participants

The demographic data of the four groups of participants, including sex, age, diabetes, hypertension, hyperlipidemia, smoking history, and drinking history, were comparable (Table 1).

**TABLE 1:** Demographic data of the study subjects.

	HC (*n* = 28)	MRP (*n* = 52)	MLP (*n* = 46)	MWP (*n* = 53)	F1	F2	P1	P2
Age(years)	35.14 ± 7.0	37.5 ± 9.8	36.7 ± 9.7	39.1 ± 7.0	1.053	0.911	0.371	0.404
Gender(Male/Female)	14/14	30/22	24/22	26/27	0.290	0.396	0.832	0.674
Diabetes	0	1 (1.8%)	3 (6.5%)	2 (3.8%)	0.912	0.673	0.437	0.512
Hypertension	0	23 (44.2%)	15 (32.6%)	18 (44.0%)	6.128	0.870	0.001	0.421
Hyperlipidemia	0	4 (7.7%)	5 (10.9%)	11 (20.8%)	3.111	2.129	0.028	0.123
Smoking	5 (17.9%)	14 (26.9%)	10 (21.7%)	15 (28.3%)	0.084	0.112	0.968	0.894
Drinking	6 (21.4%)	9 (17.3%)	9 (19.6%)	12 (22.6%)	0.165	0.232	0.920	0.793

Data presented as mean ± SD for age. One-way ANOVA, SPSS 23. F1 and P1 value is the comparison result between the four groups; F2 and P2 value is the comparison result between the three groups of MRP, MLP, and MWP.

### Overview of motion in the data

Table 2 shows the quantification of the movement parameters (measures of rotation, translation, and mean displacement in the frame-to-frame movement) of the four groups. The results shows there were no significant differences in the movement parameters between the four groups.

**TABLE 2:** Overview of motion in the data.

	HC (*n* = 28)	MRP (*n* = 52)	MLP (*n* = 46)	MWP (*n* = 53)	F	*P*
rotation (degree)	1.04 ± 0.43	1.08 ± 0.39	1.13 ± 0.38	1.25 ± 0.86	1.156	0.328
translation (mm)	1.71 ± 0.47	1.72 ± 0.39	1.83 ± 0.47	1.72 ± 0.35	0.771	0.512
mean displacement	0.90 ± 0.31	0.99 ± 0.32	1.09 ± 0.25	0.99 ± 0.34	2.157	0.095

Data presented as mean ± SD for all variables. One-way ANOVA, SPSS 23.

### Comparison of fractional anisotropy

**FIGURE 2:**
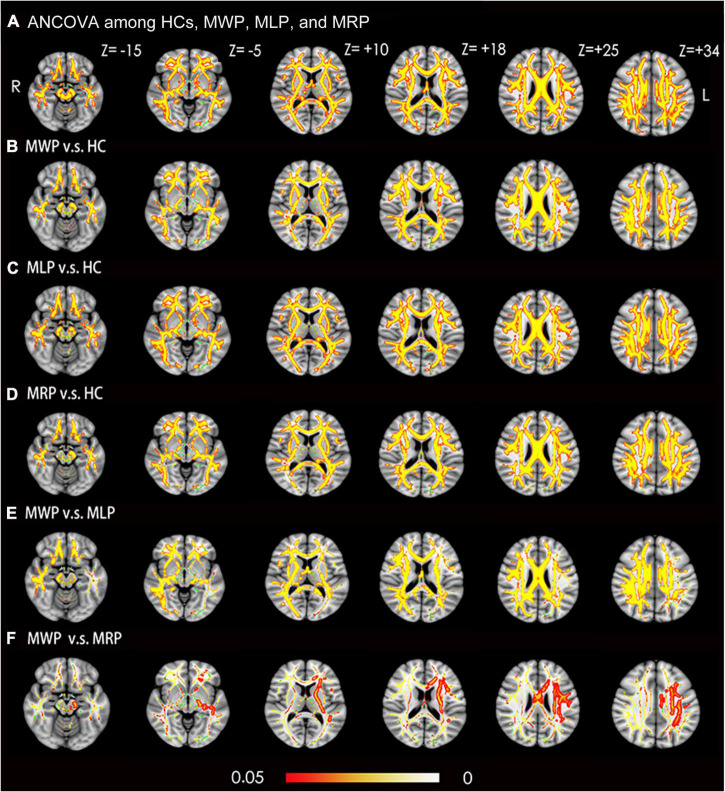
Voxel-wise TBSS analysis results of fractional anisotropy (FA) images across MMD patients with or without limb paresthesia and healthy controls (HCs). **(A)** Significant differences in FA (red-yellow) between patients without paresthesia (MWP), patients with left-limb paresthesia (MLP), patients with right-limb paresthesia (MRP), and HCs (*p* < 0.05; FWE corrected based on the threshold-free cluster enhancement statistical image). *Post hoc* analyses revealed significant reductions in FA (red-yellow) in the MWP **(B)**, MLP **(C)**, and MRP **(D)** groups in comparison with HCs. Post hoc analyses also revealed significant reductions in FA (red-yellow) in the MLP **(E)** and MRP **(F)** groups in comparison with the MWP group. Green represents the mean white matter skeleton of all participants. The color scale (red-yellow) represents significant differences between groups, with colored regions exceeding the significance threshold of *P* < 0.05. The left side of the image corresponds to the right hemisphere of the brain. ANCOVA, analysis of covariance.

Fractional anisotropy was compared among the HC, MWP, MRP, and MLP groups. In comparison with HCs, the MWP, MRP, and MLP groups showed significantly lower FA values in the following regions (Figures 2A–D): bilateral superior longitudinal fasciculus, bilateral corona radiata, body of the corpus callosum, genu of the corpus callosum, splenium of the corpus callosum, bilateral cingulum (cingulate gyrus), bilateral sagittal stratum (including inferior longitudinal fasciculus and inferior fronto-occipital fasciculus), bilateral anterior and posterior limbs of the internal capsule, bilateral external capsule, right cerebral peduncle, bilateral superior cerebellar peduncle, right fornix (cres)/stria terminalis, and bilateral posterior thalamic radiation (including the optic radiation).

In comparison with the MWP group, the MLP group showed significantly lower FA values in the following regions (Figure 2E): bilateral superior longitudinal fasciculus, bilateral corona radiata, body of the corpus callosum, genu of the corpus callosum, splenium of the corpus callosum, bilateral cingulum (cingulate gyrus), right sagittal stratum (including inferior longitudinal fasciculus and inferior fronto-occipital fasciculus), right anterior and posterior limb of internal capsule, right external capsule, right cerebral peduncle, right superior cerebellar peduncle, right fornix (cres)/stria terminalis, and right posterior thalamic radiation (including the optic radiation). Additionally, FA also showed significant reductions in the following regions in the MRP group (Figure 2F): body of the corpus callosum, left corona radiata, left anterior and posterior limb of the internal capsule, left cerebral peduncle, and left sagittal stratum (including the inferior longitudinal fasciculus and inferior fronto-occipital fasciculus).

### Comparison of mean kurtosis

**FIGURE 3:**
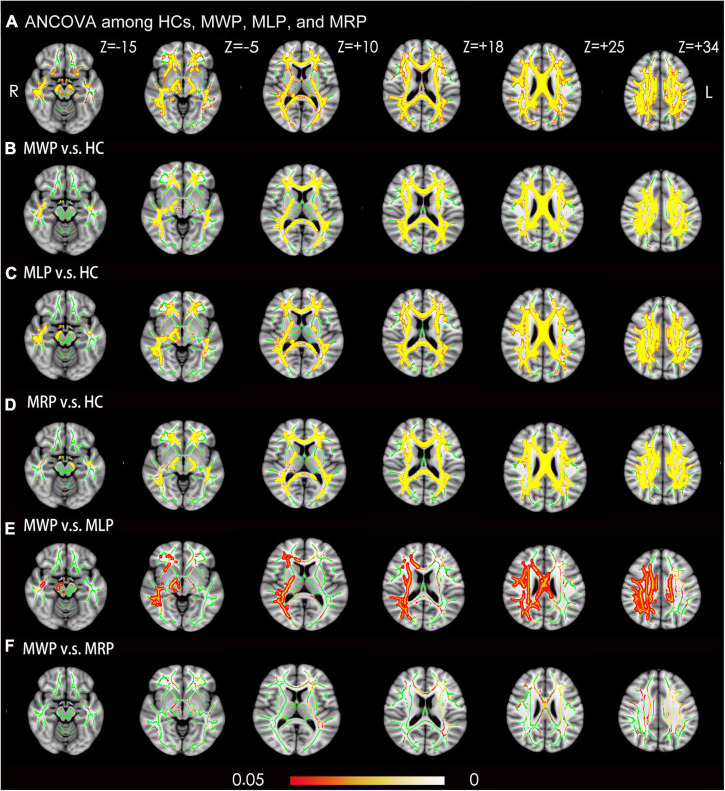
Voxel-wise TBSS analysis results of mean kurtosis (MK) images across MMD patients with or without limb paresthesia and healthy controls (HCs). **(A)** Significant differences in MK (red-yellow) between patients without acroparesthesia (MWP), patients with left-limb paresthesia (MLP), patients with right-limb paresthesia (MRP), and HCs (*p* < 0.05; family-wise error corrected based on the threshold-free cluster enhancement statistical image). *Post hoc* analyses revealed significant reductions (red-yellow) in MK in the MWP **(B)**, MLP **(C)**, and MRP **(D)** groups in comparison with the HCs. *Post hoc* analyses revealed significant reductions in MK in the MLP group **(E)** in comparison with MWP group, and no significant reductions in MK were observed in the MRP group **(F)** in comparison with the MWP group. Green represents the mean white matter skeleton of all participants. The color scale (red-yellow) represents significant differences between groups, with colored regions exceeding the significance threshold of *P* < 0.05. The left side of the image corresponds to the right hemisphere of the brain. ANCOVA, analysis of covariance.

Ean kurtosis was compared among the HC, MWP, MRP, and MLP groups. In comparison with HCs, the MWP, MRP, and MLP groups showed significantly lower MK values in the following regions (Figures 3A-D): bilateral superior longitudinal fasciculus, bilateral corona radiata, body of the corpus callosum, genu of the corpus callosum, splenium of the corpus callosum, right cingulum (cingulate gyrus), bilateral sagittal stratum (including inferior longitudinal fasciculus and inferior fronto-occipital fasciculus), bilateral posterior limb of the internal capsule, bilateral cerebral peduncle, and bilateral posterior thalamic radiation (including optic radiation).

In comparison with the MWP group, the MLP group showed a significant reduction in MK in the following regions (Figure 3E): right superior longitudinal fasciculus, right corona radiata, body of the corpus callosum, the right splenium of the corpus callosum, right cingulum (cingulate gyrus), right sagittal stratum (including the inferior longitudinal fasciculus and inferior fronto-occipital fasciculus), right posterior limb of the internal capsule, right cerebral peduncle, and right posterior thalamic radiation (including the optic radiation). The MRP and MWP groups showed no significant difference in MK values (Figure 3F).

### Comparison of global network parameters

**FIGURE 4:**
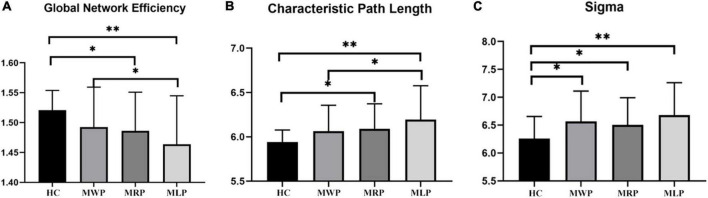
Comparison of global network parameters. In comparison with the findings for healthy controls (HCs), global network efficiency **(A)** was decreased, and characteristic path length **(B)** was increased, both in the order of MMD patients without paresthesia (MWP), patients with right-limb paresthesia (MRP), and patients with left-limb paresthesia (MLP); in contrast, sigma values increased in the order of MRP, MWP, and MLP **(C)**. The asterisk (*) denotes results without family-wise error (FWE) correction, **p < 0.05 after FWE correction, and ***p < 0.01 after FWE correction.

In comparison with HC, MMD patients showed lower global efficiencies in the following order: MWP > MRP > MLP (Figure 4A); increased characteristic path length in the following order: MLP > MRP > MWP (Figure 4B); and increased sigma values in the following order: MLP > MWP > MRP (Figure 4C). MMD patients with left-limb paresthesia showed the lowest global efficiency, longest characteristic path length, and largest sigma value.

### Comparison of local network parameters

#### Nodal efficiency

**FIGURE 5:**
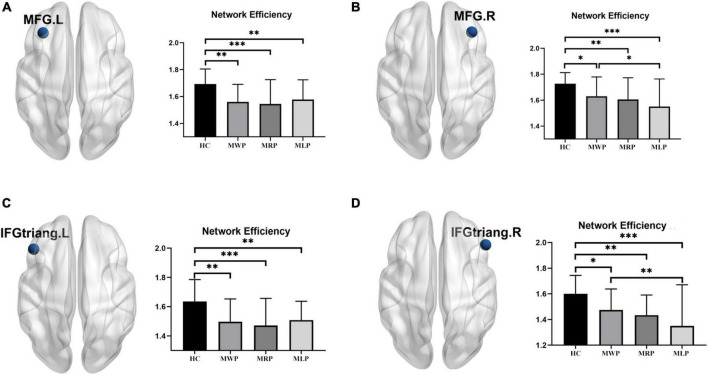
Comparison of nodal efficiency in the local network. In comparison with the healthy controls (HCs), MMD patients without paresthesia (MWP), patients with right-limb paresthesia (MRP), and patients with left-limb paresthesia (MLP) showed reductions in nodal efficiency in the following brain regions: MFG.L **(A)**: MLP > MWP > MRP; MFG.R **(B)**: MWP > MRP > MLP; IFGtriang.L **(C)**: MLP > MWP > MRP; and IFGtriang.R **(D)**:MWP > MRP > MLP;. The asterisk (*) denotes results without family-wise error (FWE) correction, **p < 0.05 after FWE correction, and ***p < 0.01 after FWE correction.

In comparisons with HCs, four of the 90 nodes showed significant differences (*p* < 0.05) in nodal efficiency, including the left middle frontal gyrus (MFG.L), right middle frontal gyrus (MFG.R), left inferior frontal gyrus pars triangularis (IFGtriang.L), and right inferior frontal gyrus pars triangularis (IFGtriang.R). The nodal efficiency of MMD patients was lower in these regions; patients with right-limb paresthesia showed the lowest nodal efficiency in MFG.L (Figure 5A) and IFGtriang.L (Figure 5C), and patients with left-limb paresthesia showed the lowest nodal efficiency in MFG.R (Figure 5B) and IFGtriang.R (Figure 5D).

#### Degree

**FIGURE 6:**
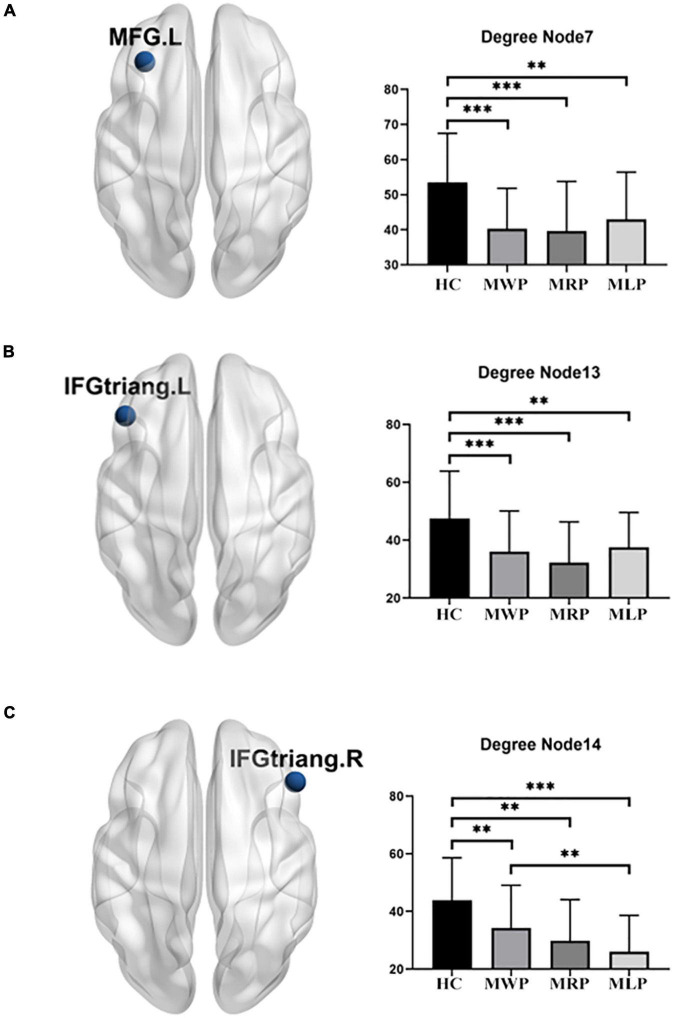
Comparison of degrees in the local network. In comparison with the healthy controls (HCs), MMD patients without paresthesia (MWP), patients with right-limb paresthesia (MRP), and patients with left-limb paresthesia (MLP) showed reductions in degree in the following brain regions: MFG.L **(A)**: MLP > MWP > MRP; IFGtriang.L **(B)**: MLP > MWP > MRP; and IFGtriang.R **(C)**: MWP > MRP > MLP. Asterisk (*) denotes results without family-wise error (FWE) correction, ^**^p < 0.05 after FWE correction, and ^***^p < 0.01 after FWE correction.

In comparisons with HCs, three of the 90 nodes (MFG.L, IFGtriang.L, and IFGtriang.R) showed significant differences in degree. The degree decreased in these regions: the patients with right-limb paresthesia showed the lowest degree in MFG.L (Figure 6A) and IFGtriang.L (Figure 6B), and the patients with left-limb paresthesia showed the lowest degree in IFGtriang.R (Figure 6C).

#### Preoperative and postoperative comparisons

At 3–4 months after EDAS, the clinical symptoms in the 35 patients with acroparesthesia had reduced or disappeared, but no significant changes were observed in FA, MK, global or local network parameters after the operation.

The 24 patients without acroparesthesia showed preoperative symptoms of dizziness, headache, blurred vision, or fluency disorder, which reduced or disappeared at 3–4 months after unilateral EDAS, but no significant changes were observed in WM structural or network parameters after the operation.

## Discussion

Diffusion kurtosis imaging is a diffusion-weighted MRI technique that requires the use of at least three b-values and 15 diffusion directions to estimate diffusional kurtosis. In early DKI studies, six b-values, ranging from 0 to 2,500 s/mm^2^ in increments of 500 s/mm^2^, were often used. The advantage of using more than three b-values is that it allows assessment of the fitting model’s goodness of fit. However, with three b-values (0, 1,000, and 2,000 s/mm^2^), the acquisition time is shorter and brain tissue changes can be evaluated more conveniently and effectively (Jensen and Helpern, 2010). Therefore, based on the tolerance level of the patient, we used a three b-value protocol for DKI to investigate the WM changes in patients with MMD.

The results of this study indicate that the changes in FA and MK derived from DKI can reflect microstructural changes in brain tissue of patients with MMD before detection of changes in the brain parenchyma by conventional MRI. In patients with MMD, the WM microstructure may change during long-term chronic ischemia, and these changes may occur even among patients with normal-appearing WM. This result is partly consistent with the observations recorded in previous studies (Hofer and Frahm, 2006; Jeong et al., 2011; Kazumata et al., 2016a). Our TBSS analysis identified widespread FA and MK reductions among patients, and the regions showing a reduction in FA were larger than those showing a reduction in MK. It seems that the change in FA value is more sensitive in detecting the change in WM microstructure, but abnormal FA can also be influenced by multiple non-biological factors (e.g., scanner parameters, data quality, head motion, and so on) and the presence of crossing fibers within a voxel (Volz et al., 2018). On the other hand, FA represents the degree of anisotropy of the diffusion of water molecules in the tissue, and MK represents the complexity or heterogeneity in the tissue microenvironment; the two values complement each other to better reflect the microstructural changes in brain tissue (Jensen and Helpern, 2010). In an experimental animal model of chronic WM ischemia, myelin sheath damage preceded axonal damage, suggesting that myelin sheath changes are the main pathological changes in the WM in cases of chronic cerebral perfusion deficiency (Kurumatani et al., 1998). Therefore, for patients with MMD, FA and MK reductions may be related to long-term chronic ischemia (O’Sullivan et al., 2001; Shiraishi et al., 2005; Jeong et al., 2011).

FA reductions reflect microstructural changes of the WM that probably indicate reduced myelination of WM tracts. On the other hand, a reduction in MK usually indicates a reduction in the complexity of fibrous tissue in these regions, which reflects microstructural changes with regard to attenuation of the myelin sheath and/or axon. Thus, our findings may reveal impairment of the myelin sheath rather than axonal loss in MMD patients (Cechetti et al., 2012; Kazumata et al., 2016a; Li et al., 2021).

Graph theory analysis of the DKI-based structural network revealed the small-world network changes in patients with MMD. In these patients, the global efficiency of information transmission in the brain network decreases with an increase in the characteristic path length, and the changes were greater in patients with acroparesthesia. The shorter the path length, the higher the global efficiency of information transmission (Latora and Marchiori, 2001; Liao et al., 2017), and global efficiency was affected by the loss of long-range connections (8). In patients with MMD, extensive WM injury during long-term chronic ischemia may cause damage to the long-range connections between brain regions, thus decreasing global efficiency.

Comparison of the local network parameters in MMD patients with acroparesthesia showed that the nodal efficiency decreased in bilateral MFG and IFGtriang, while the degree decreased in MFG.L and bilateral IFGtriang. Patients with right-limb paresthesia showed the lowest nodal efficiency and degree in MFG.L and IFGtriang.L, while patients with left-limb paresthesia showed the lowest nodal efficiency in MFG.R and IFGtriang.R and the lowest degree in IFGtriang.R. MFG is connected to the frontopolar region, supplementary motor area, premotor cortex, and IFG through intralobar short-range U-shaped tracts, forming the frontal longitudinal system as an extension of the superior longitudinal fasciculus (short frontal lobe connections of the human brain). The functions of cortical-cortical connections in these areas are largely unknown, and these connections may be involved in executive function and memory (Catani et al., 2012; Kazumata et al., 2016b). The results of our study suggested that changes in the connections of MFG, IFGtriang, and the whole brain are closely associated with the development of acroparesthesia in patients with MMD and may be an important link for evaluating disease severity in MMD patients with acroparesthesia.

Additionally, this study showed an interesting result. In comparison with the MMD patients without limb paresthesia, those with left-limb paresthesia exhibited more extensive WM injury than those with right-limb paresthesia, and the MMD patients with left-limb paresthesia showed the lowest global efficiency and the longest characteristic path length. This may be related to the fact that only right-handed patients, who were more sensitive to subtle changes in right-limb paresthesia, were recruited. Therefore, left-limb paresthesia may be detected later than right-limb paresthesia, causing more severe damage to the WM and greater changes in the structural network.

Unfortunately, in this study, although the patients’ symptoms improved or disappeared after revascularization, all three groups showed no significant differences in FA, MK, or global and local network parameters before and after surgery (MWP, MLP, and MRP). On one hand, this could be attributed to the short time interval between postoperative reexamination and preoperative examination. When hemodynamics are improved after revascularization, the microstructure of brain tissue may not easily show changes within a short period of time. On the other hand, the plasticity of brain function can help patients regain functionality neuro-elastically by rerouting functional pathways elsewhere to compensate for permanently damaged neuropathways (Klein et al., 2018; Wang et al., 2020; Xing et al., 2021). A longer-term follow-up period could facilitate the observation of changes in DKI parameters after an improvement in cerebral hemodynamics and validate our hypothesis.

## Limitations

This study had multiple limitations: (1) Limb paresthesia in the MMD patients manifested as paroxysmal limb numbness or hypoesthesia, and the grading of the symptom severity was lacking, so analysis of the correlation of this symptom with DKI results could not be performed. (2) The patients were followed up for only 3–4 months, although long-term follow-up evaluations would have been more useful to study the changes in the brain network with improved cerebral hemodynamics, because all of the patients underwent indirect revascularization. (3) In this study, we used DKI with TBSS to detect WM changes in MMD patients with limb paresthesia. However, the skeleton projection step in TBSS’s had some deficiencies in normalization strategy (Bach et al., 2014). Better registration methods, such as voxel-based analyses of DTI using groupwise registration based on ANTS or other well-performing non-linear registration algorithms, can yield more accurate registration results (Schwarz et al., 2014). (4) Because of limitations imposed by the hardware performance of the MRI scanner and the degree of patient cooperation, the spatial resolution and angular resolution of the data in this study were relatively low, which may have influenced the accurate construction of the brain network.

## Conclusion

In conclusion, DKI can detect the structural and network changes in patients with MMD. FA is more sensitive than MK in detecting WM injury in MMD patients with acroparesthesia. The global and local network parameters of a whole-brain network in patients with MMD changed with chronic ischemia, and these changes affected the clinical symptoms of the patients, MFG and IFGtriang are the key nodes related to the development of acroparesthesia. DKI can be used to assess the severity of chronic ischemic injury from a network perspective, which may facilitate disease assessment and prognostic evaluations.

## Data availability statement

The raw data supporting the conclusions of this article will be made available by the authors, without undue reservation.

## Ethics statement

The studies involving human participants were reviewed and approved by the Ethics Committee of Affiliated Hospital of Academy of Military Medical Sciences. Written informed consent to participate in this study was provided by the participants’ legal guardian/next of kin. Written informed consent was obtained from the individual(s) for the publication of any potentially identifiable images or data included in this article.

## Author contributions

RS and S-YZ designed this study, and contributed in collection, analysis and interpretation of data and drafting the article, they made the most important contribution to the study. P-GQ and G-JL contributed in the conception and design. XC and S-YZ contributed in analysis of data. All authors have made contributions to revising the article critically for important intellectual content.

## Footnotes

1
https://fsl.fmrib.ox.ac.uk/fsl

2
https://www.nitrc.org/projects/dke
